# A chart review on surgical myocardial debridging in symptomatic patients: a safe procedure with good long-term clinical outcome and coronary computed tomographic angiography results

**DOI:** 10.1093/icvts/ivac286

**Published:** 2023-01-09

**Authors:** Zohra Charaf, Kaoru Tanaka, Francis Wellens, Jan Nijs, Ines Van Loo, Jean-Francois Argacha, Mark La Meir

**Affiliations:** Department of Cardiac Surgery, UZ Brussel, Brussels, Belgium; Department of Radiology, UZ Brussel, Brussels, Belgium; Department of Cardiac Surgery, UZ Brussel, Brussels, Belgium; Department of Cardiac Surgery, UZ Brussel, Brussels, Belgium; Department of Cardiac Surgery, UZ Brussel, Brussels, Belgium; Department of Cardiology, UZ Brussel, Brussels, Belgium; Department of Cardiac Surgery, UZ Brussel, Brussels, Belgium

**Keywords:** Myocardial bridging, Coronary, Coronary computed tomographic angiography, Myotomy, Unroofing

## Abstract

**OBJECTIVES:**

Myocardial bridging is mostly diagnosed as an incidental imaging finding but can result in severe vessel compression and significant clinical adverse complications. Since there is still an ongoing debate when to propose surgical unroofing, we studied a group of patients where this was performed as an isolated procedure.

**METHODS:**

In 16 patients (38.9 ± 15.7 years, 75% men) who had surgical unroofing for symptomatic isolated myocardial bridges of the left anterior descending artery, we retrospectively analysed symptomatology, medication, imaging modalities used, operative techniques, complications and long-term outcome. Computed tomographic fractional flow reserve was calculated to understand its potential value for decision-making.

**RESULTS:**

Most procedures were performed on-pump (75%, mean cardiopulmonary bypass 56.5 ± 27.9 min, mean aortic cross-clamping 36.4 ± 19.7 min). Three patients needed a left internal mammary artery bypass since the artery dived inside the ventricle. There were no major complications or deaths. The mean follow-up was 5.5 years. Although there was a dramatic improvement in symptoms, still 31% experienced atypical chest pain at various moments during follow-up. Postoperative radiological control was performed in 88%, showing no residual compression or recurrent myocardial bridge and patent bypass if performed. All postoperative computed tomographic flow calculations (7) showed a normalization of coronary flow.

**CONCLUSIONS:**

Surgical unroofing for symptomatic isolated myocardial bridging is a safe procedure. Patient selection remains difficult but introducing standard coronary computed tomographic angiography with flow calculations could be helpful in preoperative decision-making and during follow-up.

**Figure:**
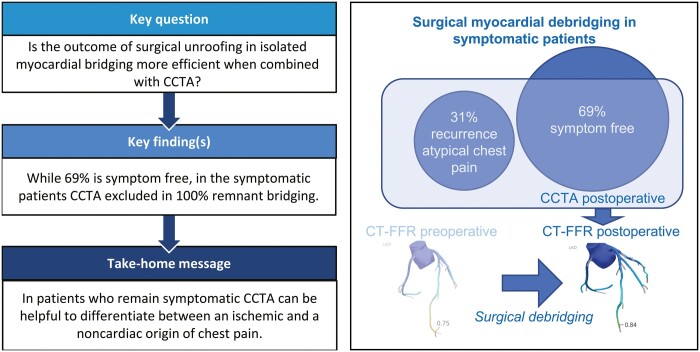

## INTRODUCTION

Myocardial bridging (MB), a congenital anomaly in which a segment of an epicardial coronary artery takes an intramuscular course, can result in vessel compression during systole. Most bridges are located in the middle segment of the left anterior descending artery (LAD) [1]. Frequently documented as asymptomatic, this compression may cause adverse complications such as angina, myocardial ischaemia [2] acute coronary syndromes [3–5], left ventricular dysfunction [6], arrhythmias [7, 8] and sudden cardiac death [9, 10]. Incidence and prevalence statistics vary widely in literature, due to variation in definitions used and methods of detection. In a recent meta-analysis by Roberts *et al.* [11], MB is found in 4.3% of patients undergoing coronary angiography (CA), in 21.7% of coronary computed tomographic angiography (CCTA), depending on types of computed tomography (CT) and in- or exclusion of superficial MBs and in 44.6% in autopsy dissections. Hostiuc *et al.* [12] suggested that in evaluating prevalence, high-resolution CT scanning should be preferred to CA. Since there is still an ongoing debate concerning the clinical significance of MB and the link between symptoms and the mechanism of pathophysiology [13], most patients will be treated conservatively. Therefore, it is important to better understand who will benefit from an intervention. In symptomatic patients refractory to medical therapy, surgical unroofing by dividing the myocardium overlying the epicardial coronary artery is the only therapeutic option directly treating the underlying pathology.

We present our institutional experience of surgical unroofing of MB in symptomatic patients with no concomitant surgical treatment, with particular attention to long-term clinical outcome and postoperative follow-up using CT imaging modalities.

## PATIENTS AND METHODS

### Ethical statement

The study was approved by our Institutional Ethical Commission of the University Hospital Brussels on 22 April 2022 (Number 2022-100). Written informed consent was not required. The patient group was searched in our database of surgery schedule, from July 2011 to July 2020.

A group of 25 patients with the diagnosis of symptomatic MB were referred to the HeartTeam and were proposed surgery. Three patients went for second and third opinions to other hospitals. In 2 cases, they opted for non-operative management. The third patient was operated in another institution.

To highlight the exclusive potential benefit of debridging, we excluded 6 patients with the concomitant performance of other surgeries [other coronary artery diseases for which simultaneous bypass grafting (2), aortic valve replacement (1), reimplantation of abnormal right coronary (2), morrow procedure (1)].

### Patient population

Sixteen patients, 12 refractory to medical treatment with beta-blockers or calcium-channel blockers for at least 1 month, were included over a period of 9 years. One patient did not tolerate medical therapy due to hypotension, 2 due to bradycardia and 1 professional athlete refused medical therapy. The majority of patients were men (75%), and the mean age at time of operation was 38.9 ± 15.7 years. None of the patients were lost to follow-up. Our patient population mainly consisted of young and physically active individuals (recreational or professional). Retrosternal pain was present in 14 patients at the time of diagnosis and typically provoked during sport activities in 93%. Two patients presented with acute coronary syndrome with elevated troponins (500 and 522 µg/l). All other symptoms at diagnosis are shown in Table 1.

**Table 1:** Symptoms at diagnosis

Symptom	Frequency, *n* (%)
	Total *n* = 16
Chest pain^a^	14 (88)
Grade I	2 (14)
Grade II	3 (21)
Grade III	3 (21)
Grade IV	6 (43)
Dyspnoea	6 (38)
Palpitations	4 (25)
Syncope	5 (31)
Ventricular arrythmia	0 (0)

a Chest pain was classified by the Canadian Cardiovascular Society Angina Classification System.

The preoperative echocardiography was without dobutamine and showed no typical septal buckling with apical sparing, cardiac function was normal. Five patients had a positive cyclo-ergometry test in which ischaemia signs were observed, 7 patients stopped the test early due to retrosternal pain and 3 patients did not undergo a cyclo-ergometry test seen ST-depression in rest, positive troponins during symptoms or referred with a positive myocardial perfusion scan.

**Figure 1:**
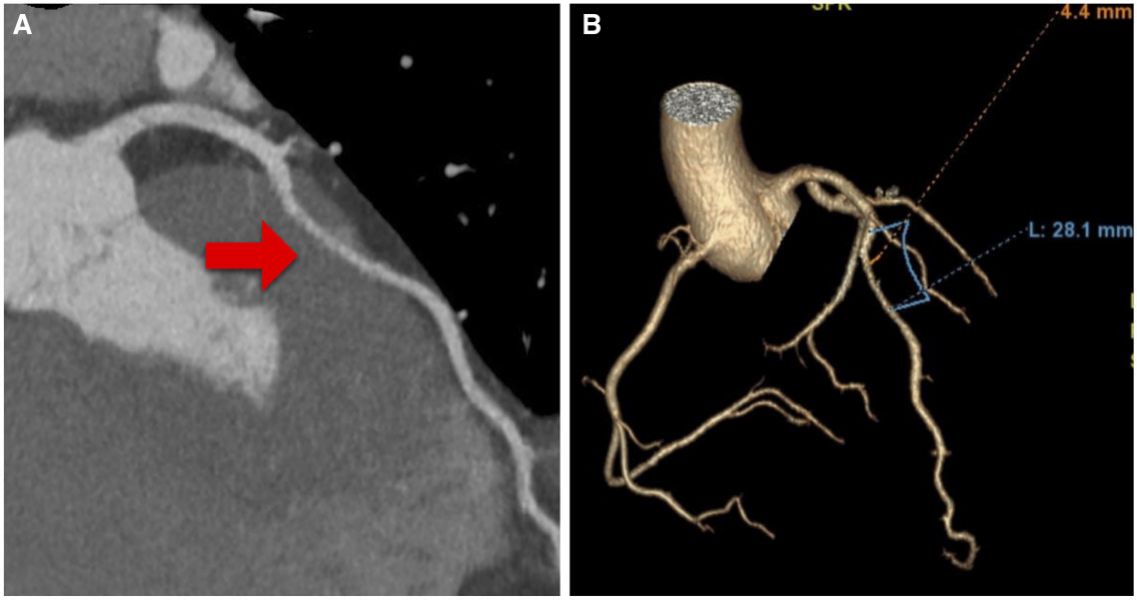
(**A**) Coronary computed tomographic angiography showing a myocardial bridge of the mid-left anterior descending artery (arrow), length 28.1 mm and depth 4.4 mm. (**B**) Three-dimensional reconstruction.

Six patients underwent a CCTA as first additional imaging, diagnosing an MB of the LAD and demonstrating the exact location, length and depth of the bridge (Fig. 1). One patient underwent a CCTA after the CA because high suspicion of right ventricle (RV) entering of the LAD. Another patient came for a second opinion, and CA was already performed, so additional CCTA was performed in our institute. Since July 2015, all our patients received a CCTA preoperatively, as CCTA became more accessible. The mean length of the MB on CCTA was 29.0 ± 12.2 mm and the mean depth of 3.6 ± 1.1 mm. The summary of findings of preoperative imaging with CCTA is given in Supplementary Material, Table S2.

**Figure 2:**
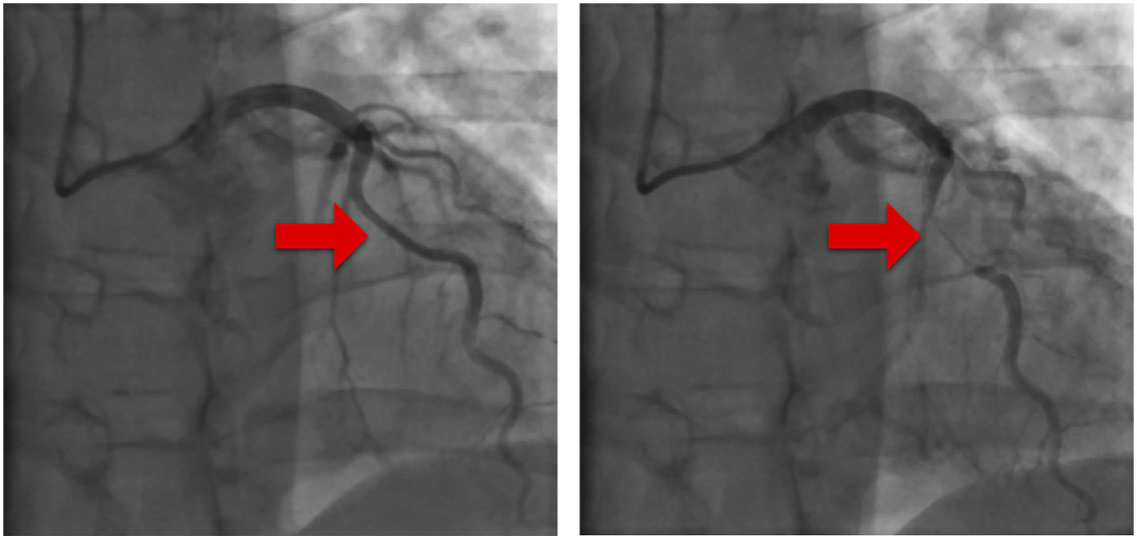
Total occlusion of mid-left anterior descending artery (arrow) during systole on coronary angiography.

All patients underwent CA, which confirmed mid-LAD bridging (Fig. 2), with mean length 26.2 ± 10.0 mm (minimum 16.4 mm, maximum 52.2 mm) and mean percentage milking of 65% (minimum 48%, maximum 93%). None of them showed significant stenosis proximal to the MB. Further CA characteristics are listed in Supplementary Material, Table S3. One patient’s images were not available.

### Operative technique

**Figure 3:**
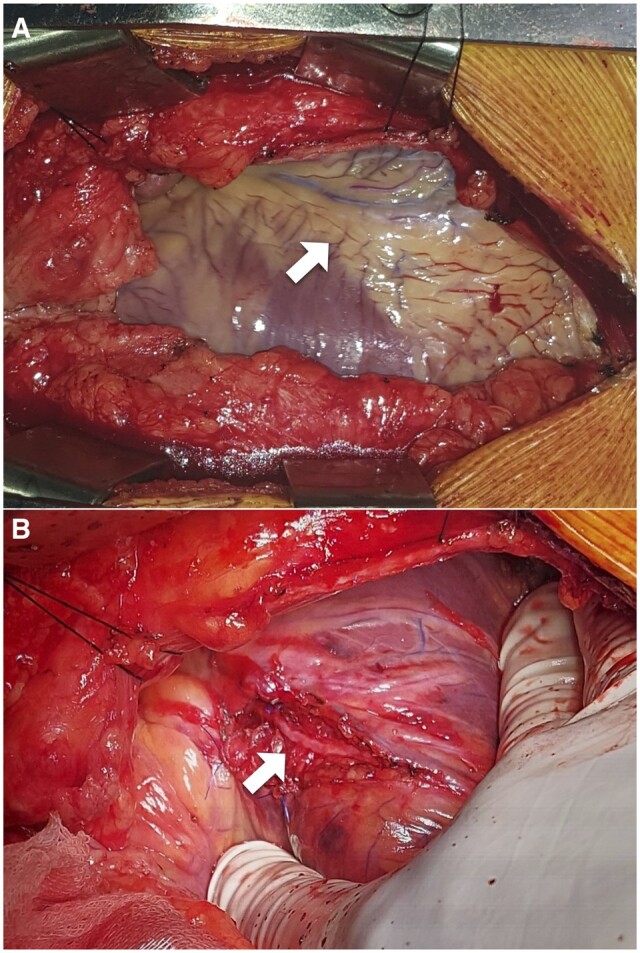
(**A**) Bridged segment of mid-left anterior descending artery during off-pump procedure (arrow). (**B**) Left anterior descending artery after unroofing the overlying myocardium (arrow)

All procedures were performed through median sternotomy, mostly (75%) with cardiopulmonary bypass (CPB), aortic cross-clamping and cardioplegic arrest. The mean CPB time was 56.5 ± 27.9 min, and aortic cross-clamping time was 36.4 ± 19.7 min. The remaining patients had an off-pump procedure. After identification of the LAD distal to the MB, its overlying myocardium was carefully dissected with a Beaver^®^ blade knife from distal to proximal (Fig. 3). If after anterior debridging, the LAD was still imbedded into the muscle by its lateral walls, and a marsupialization of the muscle was made. This technique consisted of retracting the adjacent muscle layer to respectively left and right with separated suture points of a monofilament non-absorbable suture.

Intraoperative transoesophageal echocardiography was used to evaluate ventricular function.

## RESULTS

### Statistical analysis

Data were retrospectively entered into a database. Normally distributed continuous variables are expressed as mean ± standard deviation, unless otherwise specified. Categorical variables are expressed as numbers and percentages.

### Intraoperative

Six patients (38%) were planned for off-pump surgery, 2 were converted to CPB since a deep course of the LAD into the RV or septum was observed. In the first patient, the RV was entered laterally and medially of the LAD; in the second patient, the LAD had a deep intraseptal course unclear on preoperative imaging and myocardial dissection had to be stopped at an early stage. The RV perforation was repaired with a monofilament non-absorbable suture. These patients needed an additional coronary bypass using the left internal mammary artery (LIMA) because of incomplete unroofing of the LAD.

A patient with prior percutaneous coronary intervention of the proximal LAD had a fractional flow reserve proximal to the bridge of 0.80 and distal to the bridge of 0.69; therefore, after complete debridging, a coronary bypass with LIMA was made. In 2 other cases, RV was entered during dissection. Debridging was completed and the opening was repaired with a monofilament non-absorbable suture or with a small pericardial patch. No iatrogenic injury was made to the LAD.

In 38% of the cases, a marsupialization of the adjacent muscle to the LAD was performed to avoid lateral compression. In 1 case, the LAD overlying myocardium was extremely extended and included also the 2 diagonal branches.

### Early postoperative

The median length of hospital stay was 6 days (minimum 5 days, maximum 10 days); the median length of intensive care unit stay was 1 day (maximum 2 days). All patients were extubated within 8 h of operation (mean 4.3 ± 1.8 h). There were no deaths. Atrial fibrillation occurred in 1 patient, which was chemically converted with Amiodarone^®^ within 24 h, without recurrence during entire follow-up. Two patients had clinical and electrocardiographically signs of pericarditis, successfully treated with high-dose Aspirin^®^ and Colchicine^®^. A complete summery of peri- and postoperative complications is given in Table 2.

**Table 2:** Per- and postoperative complications

	Frequency, *n* (%)
	Total *n* = 16
**Complications perioperative**	
Right ventricle entering	5 (31)
LAD injury	0 (0)
Additional bypass for incomplete dissection	3 (19)
**Complications postoperative**	
Re-operation for bleeding	0 (0)
Atrial fibrillation	1 (6)
Pneumothorax	1 (6)
**Conservative management**	
Wound infection	0 (0)
Pericarditis	2 (13)
Acute on chronic renal dysfunction	1 (6)
**Spontaneous recuperation**	
Myocardial infarctions	0 (0)

LAD: left anterior descending artery.

We saw that medication, induced for symptom relief, could be stopped postoperatively. There were 5 patients who continued a beta-blocker due to hypertension or sinustachycardia, 1 patient continued a calcium-channel blocker due to hypertension.

### Follow-up

During follow-up (median 5.8 years; ranging from 20 to 108 months), no major adverse cardiac events or death were documented. All patients were able to resume their physical activity. Annual follow-up consisted of electrocardiogram, ultrasounds and cyclo-ergometry.

Seven patients (44%) had a routine control CCTA during follow-up (1 month, 3 months, 8 months, 1 year or 5 years). There was no remnant bridge and in 2 cases where a bypass was performed, it showed a properly patent bypass (at 8-month follow-up and at 2-year follow-up).

In case of abnormal results during routine control, presentation of atypical chest pain or other major symptoms, an additional CCTA or CA was performed.

Eight patients underwent additional imaging, 6 CCTAs and 2 CAs.

- Five patients (31%) consulted with atypical retrosternal pain, occurring at non-specific moments during follow-up (at 9 months and 2–6 years). Additional CCTA controls were performed to rule out any cardiac cause, to check the efficacy of the debridging, the patency of the arterial bypass or the presence of other coronary artery diseases. All results were negative; there were no signs of coronary calcification or stenosis and in case of coronary artery bypass graft (CABG), the bypass was patent.
- One patient had asymptomatic ST elevations during cycling test at the second-year routine follow-up. Since CCTA was inconclusive due to movement artefacts, an additional CA was made and was negative for remnant MB. Control cyclo-ergometry at 3- and 4-year follow-up was normal and the patient remained asymptomatic.
- One patient underwent a control CA for repetitive syncopes, which showed normal coronary arteries and no bridging. Further work up of the electrophysiologists with Ajmaline testing was negative for arrhythmias. A reveal was implanted but never documented an arrhythmia even when subjective symptoms were present. After extensive investigation and exclusion of other causes, the syncopes were related to hyperventilation.
- Another patient had symptomatic repolarization disturbance during cycling test at 2-year follow-up. A control CA showed no significant coronary narrowing [resting full-cycle ratio (RFR) LAD 0.96 and RFR D1 0.95 (cut-off RFR 0.89)]. Further investigation with gastroscopy revealed reflux esophagitis grade B and antritis. This was treated successfully with proton pomp inhibitors. During further follow-up, the patient remained asymptomatic.

### Computed tomographic fractional flow reserve

Image qualities of all performed CTTAs (9 preoperative and 12 postoperative) were retrospectively reviewed and sent to HeartFlow Inc. (Redwood City, CA, USA) for computed tomographic fractional flow reserve (CT-FFR) calculation, although CT-FFR-based assessment of MB has not been validated by HeartFlow. Six preoperative cases and 7 postoperative cases have been analysed. Three preoperative CT-FFR showed significant drop in distal LAD, cut-off ≤0.80 (0.75, 0.76 and 0.80) and 3 showed no significant CT-FFR value (0.81, 0.85 and 0.87), although in these cases, the distal LAD was too small to calculate CT-FFR. All 7 postoperative CT-FFR showed negative CT-FFR in distal LAD (0.81–0.92).

**Figure 4:**
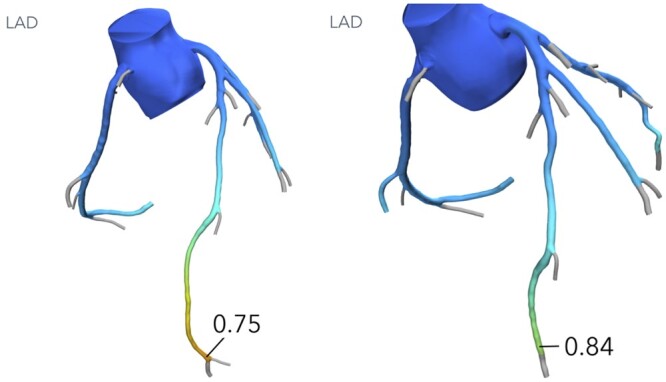
Computed tomographic fractional flow reserve 0.75 preoperative (left) and postoperative computed tomographic fractional flow reserve 0.84 (right).

An example of a pre- and postoperative CT-FFR of the LAD is demonstrated in Fig. 4.

## DISCUSSION

There is a general accepted management algorithm for the treatment of MB. In asymptomatic patients, mostly superficial MBs (<2 mm), therapeutic focus is primarily on risk factor modification. On the contrary, in symptomatic MBs (mostly deeper than 2 mm or longer than 25 mm), MBs with objective signs of ischaemia and/or abnormal intracoronary haemodynamics, medical treatment should be initiated with beta-blockers and/or calcium-channel blockers. If symptoms persist, an interventional option should be considered. In a meta-analysis of 2017 by Cerrato *et al.* [14], comparing 18 studies, the authors found that surgery was more effective than stenting in patients not responding to medical therapy. This was reconfirmed in 2020 by Murtaza *et al.* [15]. In a state-of-the-art review, Sternheim *et al.* [16] suggested percutaneous coronary intervention in shorter and more superficial MBs. Surgical revascularization with myotomy or CABG should be preferred in deep and long MBs. According to this principle, in our series, we only performed unroofing in 8 patients with a preoperative CCTA diagnosed MB deeper than 2 mm.

Surgical treatment options include supra-arterial myotomy (unroofing, debridging) or CABG, these procedures can be performed on- or off-pump, by sternotomy or mini-thoracotomy. According to Wang *et al.* [17] off-pump procedures show lower ventilator times, less total hospital stay and less blood transfusions, with no difference in morbidity, mortality or clinical outcome evaluated with the Seattle Angina Questionnaire (SAQ). CABG may be preferred when MBs are >2.5 cm long or 5 mm thick, given a theoretical concern for complications during unroofing [18]. The choice of technique will mostly depend on the surgeon’s experience [17].

There exists concern of graft failure when performing CABG for MB. Graft occlusion seems higher with LIMA than with saphenous graft, presumably because of competitive flow [16, 19]. Huang *et al.* [20] presented in their cohort of 11 patients, successful CABG with LIMA-LAD in 73%. Our study shows 3 successful LIMA grafting of whom 2 had control imaging which showed a patent graft; the third case never experienced anginal symptoms nor ischaemic test results during a total follow-up of 72 months.

The largest series of surgically treated MBs in the literature to date was published by Boyd *et al.* [19] including 50 patients with isolated symptomatic and haemodynamical significant MBs. The patients completed before and after surgery the SAQ, with significant improvements in all 5 categories: physical limitation due to angina; anginal stability; anginal frequency; treatment satisfaction; and quality of life [21]. In our population, we found that 31% of the patients represented with atypical chest pain after surgical unroofing at various moments during follow-up. All patients are currently symptom free and in NYHA Class I. Other reports also describe variable percentages of recurrent chest pain (0% [22, 23], 19% [20], up to 60% [24]). Hemmati *et al.* [24] concluded that surgical unroofing is a safe option for patients with isolated MB, but patients should be aware of the possibility of persisting or recurrent chest pain and continued need for postoperative medications despite relief of coronary compression. To better study symptomatic relief, treatment satisfaction and an overall improvement in quality of life after surgical unroofing the SAQ or shortened seven-item SAQ (SAQ-7) should be used [17, 19, 25].

There are no controlled, prospective multicentre trials using standardized postoperative haemodynamic testing after surgical treatment of MBs. Wu *et al.* [23] presented a cohort of 31 patients who underwent surgical treatment, through myotomy (16) or CABG (15), for MB of the LAD. Follow-up angiographic studies in 21 patients (68%) demonstrated restoration of coronary blood flow and myocardial perfusion without significant residual compression. Where CABG was performed the graft was patent. Xu *et al.* [22] performed postoperative imaging in their cohort of 26 patients (25 myotomies, 1 CABG) using invasive control angiography in 12 patients and CCTA in 4 patients showing in all cases complete relief of the bridge. In 88% of our patients, radiological control after surgical unroofing (12 CCTAs and 2 CAs) was performed. All controls demonstrated the normalization of coronary blood flow in the LAD. There was no residual compression or recurrent myocardial bridge and, if performed, the bypass was patent with good distal flow.

CCTA shows anatomical views and the degree of encasement of the bridged artery and assesses if bridging is likely to cause dynamic compression that would be clinical significant [26]. Also, to guide treatment strategies, detecting haemodynamically significant MBs is of clinical interest [27]. Compared to invasive fractional flow reserve, machine learning-based CT-FFR has recently shown high diagnostic performance for identifying functional ischaemia in vessels with MB [28]. Recently, Yu *et al.* [29] demonstrated that ΔCT-FFR_systolic_ (the change in CT-FFR across MB, calculated in the best systolic phase) can reliably exclude MB-related ischaemia with high sensitivity (91.7%) and negative predictive value (97.8%). In our study, we saw 3 preoperative CT-FFR with significant drop in the distal LAD, in accordance with the invasive fractional flow reserve and milking effect (all ≥80%) on CA. Routine CCTA could provide important preoperative anatomic information about length and depth of the MB and perhaps predict a successful procedure. Therefore, we would recommend the use of CCTA and CT-FFR in preoperative settings, based upon our first promising results and Yu *et al.* We also recommend the use of CT imaging modalities during follow-up to confirm unroofing efficacy and exclude any other cardiac causes in case of recurrent atypical chest pain.

### Limitations

Although similar in size to most cohorts that have been published, a major limitation of this study is a small patient population over a 9-year period. CCTAs (pre- and postoperative) were not done as standard imaging but could add to the patient selection, appropriate preoperative decision-making and postoperative follow-up. During the course of the study, imaging quality of CCTAs has dramatically improved, so not all available CCTAs were suitable for CT-FFR. Better quality could have helped us to understand the value of CCTA with CT-FFR in this patient group. Seen the difficulty to correlate imaging findings to the presence of symptoms and patient’s quality of life with the recurrence of bridging, it is important to prospectively evaluate the patient during follow-up with standardized questionnaires, such as SAQ, at regular intervals.

## CONCLUSION

There are no conclusive guidelines concerning the treatment strategy of symptomatic myocardial bridges, and larger studies are needed to define best practice [30]. Our study confirms that surgical unroofing, on- or off-pump, although not always technically easy, is a safe procedure for patients with symptomatic isolated MB who are refractory to medical treatment. Patient selection remains the most critical part, since patients could show recurrence of chest pain, despite any proven presence of myocardial ischaemia after surgical intervention. We suggest introducing standard CCTA imaging in preoperative decision-making and during long-term follow-up of those patients who remain symptomatic. This facilitates the differential diagnosis between a cardiac and noncardiac cause. To date CT-FFR is still experimental for this pathology. Although of promising value, it could be helpful to understand better the natural history to determine treatment strategy and patient selection.

## Supplementary Material

ivac286_Supplementary_Data

## Data Availability

The data underlying this article will be shared on reasonable request to the corresponding author.

## ABBREVIATIONS

CA: Coronary angiography
CABG: Coronary artery bypass graft
CCTA: Coronary computed tomographic angiography
CPB: Cardiopulmonary bypass
CT: Computed tomography
CT-FFR: Computed tomographic fractional flow reserve
LAD: Left anterior descending artery
LIMA: Left internal mammary artery
MBs: Myocardial bridgings
RFR: Resting full-cycle ratio
RV: Right ventricle
SAQ: Seattle Angina Questionnaire
