# Supplementary material for: A chart review on surgical myocardial debridging in symptomatic patients: a safe procedure with good long-term clinical outcome and coronary computed tomographic angiography results
Source: Interdiscip Cardiovasc Thorac Surg. 2023 Jan 9;36(1):ivac286. doi: 10.1093/icvts/ivac286 (PMC9931072; doi:10.1093/icvts/ivac286)
Supplement: ivac286_Supplementary_Data [file ivac286_supplementary_data.docx]

**Supplemental appendix**

Table 2. Characteristics on preoperative CCTA^a^

| **Location** | **Length (mm)** | **Depth (mm)** | **Preoperative CT-FFR^b^** |
| --- | --- | --- | --- |
| Mid-LAD | 34 | 2.7 | 0.81 |
| Mid-LAD | 22 | 5.5 | 0.85 |
| Mid-LAD | 25 | 2.4 | Not performed |
| Mid-LAD | 20 | 4 | 0.87 |
| Mid-LAD | 25 | 4 | Not performed |
| Mid, distal-LAD | 57 | 3.7 | 0.76 |
| Mid-LAD | 21 | 2.3 | 0.75 |
| Mid-LAD | 28 | 4.4 | 0.80 |

^a^Coronary computed tomographic angiography

^b^Computed tomography-Fractional Flow Reserve

Table 3. Characteristics on preoperative coronary angiography.

| **Location** | **Milking^a^ (%)** | **Length (mm)** | **iFFR^b^** |
| --- | --- | --- | --- |
| Mid-LAD | 48 | 19.8 | <0.8 |
| Mid-LAD | 84 | 16.4 | Not performed |
| Mid-LAD | 50 | 30.0 | 0.79 |
| Mid-LAD | 80 | 33.3 | 0.82 |
| Mid-LAD | 91 | 52.2 | 0.82 |
| Mid-LAD | 60 | 21.9 | 0.78 |
| Mid-LAD | 81 | 18.3 | Not performed |
| Distal-LAD | 64 | 20.2 | 0.69 |
| Distal-LAD | 54 | 31.3 | 0.62 |
| Distal-LAD | 55 | 27.3 | 0.81 |
| Mid-LAD , D1 | 93 | 18.1 | Not performed |
| Mid-LAD | 85 | 27.1 | Not performed |
| Mid, distal LAD | 87 | 39.7 | 0.89 |
| Mid-LAD | 79 | 16.5 | Not performed |
| Mid-LAD | 80 | 21.1 | Not performed |

Data is missing for one patient.

**^a^**Quantitative coronary angiography of milking is measured as loss of lumen area during systole, with OsiriX Lite, Pixmeo (Bernex, Switzerland)

**^b^**Invasive Fractional Flow Reserve
